# Cutting It Out

**Published:** 1911-02

**Authors:** 


### Cutting it Out.

Of all the campaigns which medical science has waged upon the Demon Rum, none can compare for neatness, simplicity or despatch with that which is chronicled in a recent issue of the TEXAS MEDICAL JOURNAL. With scant regard for the traditional aids to sobriety, such as the gold cure, hypnosis, doctored coffee and the dull, dark dock, the proud sponsor of this newer regeneration has declared himself enthusiastically in favor of the kill-or-cure treatment, and has started after the demon with scalpel and axe. That he has the courage of his convictions is shown by the fact that he has already performed seventeen major operations while hunting the monster to its lair.

His system of attack is based upon the conviction that the abnormal appetite for intoxicating liquor comes from a diseased condition of a section of the middle division of the small intestine situated between the duodenum and the ileum. Remove this section and presto! the appetite for liquor is gone. We are not informed whether the appetite for anything else remains or whether the patient is too far gone to partake either of solid or liquid refreshment.

At this distance from the scene of the seventeen operations we are naturally slow to pass judgment on the Texas modus operandi, but we are inclined to the view that the plan is not sweeping enough. Why stop at a section of the middle division of the small intestine? That is only a beginning. Without a digestive tract any little danger of a relapse into alcoholism would be beautifully obviated, and we would therefore respectfully suggest that while carving out the small intestine the skillful surgeon should make the most of his opportunity and take out every other part that might assist in the future assimilation of the wine while it was red.

In view of the success of the new cure, we offer this suggestion with much diffidence. For that it is successful there can be no question. In two cases at least the triumph of modern surgery over alcohol must be admitted to have been established; only fifteen of the seventeen operated upon survived.—*N. Y. Sun.*

